# Case of Artificial Anus, Arising from Ulceration of the Transverse Arch of the Colon; Which, after Discharging Feces for Eighty-One Days, Spontaneously Closed

**Published:** 1828-08

**Authors:** Edward Swarbreck Hall

**Affiliations:** Member of the Royal College of Surgeons, London; Surgeon to the South Dispensary, Liverpool.


### ARTIFICIAL ANUS.

*Case of Artificial Anus, arising from Ulceration of the Transverse Arch of the Colon; which, after discharging Feces for eighty-one days, spontaneously closed.* By EDWARD SWARBRECK HALL, Member of the Royal College of Surgeons, London; Surgeon to the South Dispensary, Liverpool.

THE subject of this case was an active, volatile girl, eleven years old. My attendance upon her commenced on the 13th October, 1827. She had then been ill six days. Her indisposition came on with pains in the belly, vomiting, loss of appetite, thirst, and fever. At the time of my visit, she was suffering from the usual symptoms of peritonitis: diffused pain over the abdomen, increased upon pressure; moist, furred tongue; dry skin; pulse 120, and weak. She was also considerably emaciated. The application of a few leeches, together with fomentations and a little diaphoretic medicine, relieved her very much. Two days afterwards, the symptoms recurred with additional violence, but were easily subdued by a small general bleeding, a blister over the abdomen, and small doses of Tincture of Colchicum with sweet Spirits of Nitre. On the 19th instant, I ceased attending my patient, as she was well nigh recovered, and her parents were afraid of incurring much expense.

On the 29th instant, I was again sent for. I found ascites had taken place; some pain of the belly had returned, with palpitation of the heart, and a pulse up to 140, but unresisting. Local blood-letting was again resorted to, and the Tinctures of Colchicum and Digitalis, in small doses gradually increased, prescribed. By the 12th of November, the dropsical symptoms had all disappeared. For some days antecedent to that date, the bowels had not performed their functions with regularity, which I attributed at that time to their having been the outlet by which the greater part of the dropsical effusion had been carried off; neither the kidneys nor skin acting with more than their usual energy. At times, even after the dropsy of the belly was dissipated, the dejections were so numerous that my patient was unable to rest at night. They were exceedingly offensive, of a whitish colour, and sometimes had very much the appearance of yeast. I have now little doubt that the mucous glands of the intestines were diseased; and probably the enlargement of the mesenteric glands, which was subsequently developed, arose from that source. The pulse was never below 120 for three months, and the tongue was more or less furred for the same period.

At this time (12th November,) I commenced the use of small doses of bluepill, which was persisted in until my patient became convalescent; sometimes combining it with narcotics, and at others with gentle purgatives, such as rhubarb. In fact, my treatment throughout this complaint was entirely on the principles of one of my much respected teachers, Mr. Abernethy, a gentleman who will ever be remembered with gratitude and respect by those who have had the honour of being his pupils. "You have no right to dictate to nature," he would say: "sooth irritation, aid her in her efforts, but do not attempt to control her."

On the 30th of November, my attention was directed to a red, prominent, and painful swelling, of about the size of a pigeon's egg, immediately below the umbilicus. It resembled a furuncle in some measure, wanting, however, the characteristic hardness of a swelling of that nature, and having rather an elastic feel, as if distended with air. The whole belly likewise was somewhat tumid and elastic; the legs were slightly œdematous, the pulse considerably accelerated, and thirst very much complained of. I ordered the tumor to be poulticed, and two tablespoonfuls of a saline julep to be taken every two hours.

At my next visit, the tumor presented a very singular appearance: the superincumbent cuticle was raised from the cutis, like a blister, but was quite elastic, and evidently distended with air. I made a very small puncture in it with my lancet: a pretty smart report followed, and very fetid air, having the odour of the gas usually discharged per anum, escaped. After this had ceased to issue, a small quantity of fecal matter exuded from the aperture. From the appearance of the fecal matter, odour of the liberated gas, and situation of the opening, I had no doubt it communicated with the transverse arch of the colon. It is most probable that, during the early part of the complaint, the colon was united to the parietes of the abdomen by adhesive inflammation, and that then ulceration commenced in one of the diseased mucous glands, and gradually extended through the coats of the intestine and walls of the abdomen. Indeed, I remember that at one time, during the existence of the diffused pain over the abdomen, she complained of it being more severe in the neighbourhood of the umbilicus than any where else, which induced me to examine that region very attentively; but I did not discover any hardness or tumefaction on that occasion. At a later inspection (13th January) I could trace very distinctly an irregular induration in that situation, which I believed to be in the meso colon.

Next day (December 2d,) I found my patient something easier: her pulse was less furred, pulse not so rapid, and thirst abated. She had passed several evacuations per anum since my last visit. Upon removing the dressings from the belly, gas again escaped. I found the cuticle which had been raised entirely detached, and beneath it in the cutis two points of ulceration, not exceeding the magnitude of pins' heads: feces slowly issued through them.

From this time until the artificial anus got well, poultices were applied every two hours. Various tonics were prescribed during the further progress of the disease, particularly Sulphate of Quinine; at the same time continuing the use of the bluepill. Generous diet was allowed her, and wine, ale, and porter freely administered. Of course, particular symptoms were prescribed for as they arose.

It would swell my communication too much to relate the daily changes that took place from this time forwards: suffice it to give a condensation of the most important ones.—The apertures in the abdomen slowly enlarged as the disease advanced, until they would admit a moderate-sized goosequill, but never extended beyond that magnitude. The surrounding integuments always preserved a healthy aspect. The discharge from the artificial anus varied exceedingly: sometimes none would take place for several days, and at others it would be very profuse, probably a quart or more in twenty-four hours. Its consistence, odour, and appearance, were also subject to variation: on one occasion, when my little patient was so bad that I apprehended her almost immediate dissolution, the smell was distinctly gangrenous; at other times it had a musty, disagreeable odour; but the highly feculent smell was most prevalent. She was frequently troubled with colicky pains, but never to any great extent, and generally previous to a dejection per anum. The anal evacuations were as mutable as those from the artificial openings, and pretty generally in an inverse ratio: that is, when the one was profuse and watery, the other would be trifling and consistent. The pulse sometimes rose as high as 160 beats in a minute, but was always weak and unresisting; and, as I have before stated, was never below 120, until the 30th January, at which time some amendment of my patient's health began to take place. The anasarca of the extremities became so great, that I was afraid the integuments would have sloughed; and it was not until her recovery was considerably advanced that it began to diminish. She was often affected with hysterical symptoms, inability to retain the urine, cough, and difficulty of deglutition. For several days, she was reduced to such an excessive state of debility that she could not articulate or move herself in bed. Her appetite was at all times exceedingly capricious, being at one period voracious, at another she subsisted for days together merely on fluids. She was likewise very often subject to vomiting, and for many days nothing would remain on her stomach but a little cold ale.

On the 30th January, a decided amelioration of her symptoms took place, and from that date she continued to improve. On the 13th February she was able to leave her bed, to which she had been confined for the protracted period of three months. The artificial anus ceased discharging on the 19th of the same month, and in eight days no aperture whatever could be detected; a puckered depression, that would admit the point of the finger, existing in its site. Her appetite had lost its fickle character, and any kind of food agreed with her. The bowels had completely resumed their healthy functions, and the evacuations were perfectly natural. Up to the present time she has remained quite well, with the exception of occasional slight pains in the belly. Her abdomen is still rather protuberant, and irregular indurations, as if of enlarged mesenteric glands, can be detected. Should she not have a daily evacuation from the bowels, which she commonly has, she feels some uneasiness, and the site of the artificial anus, instead of being depressed, becomes pouting.

Ulceration of the walls of the intestines, followed by the effusion of their contents into the cavity of the abdomen, and thereby destroying the patient, is not an uncommon consequence of disease in the mucous membrane of the bowels: but I am not aware that a case similar to that which I have related has ever been recorded. There are two cases in Mr. Howship's work on the Intestines which bear some relation to it, but they are by no means parallel.

*Liverpool; June 18th,* 1828.

